# The contribution of Lynch syndrome to early onset malignancy in Ireland

**DOI:** 10.1186/s12885-021-08263-z

**Published:** 2021-05-26

**Authors:** Alice Talbot, Emily O’Donovan, Eileen Berkley, Carmel Nolan, Roisin Clarke, David Gallagher

**Affiliations:** 1 The Royal College of Surgeons of Ireland (RCSI), Dublin, Ireland; 2 Department of Cancer Genetics, St. James’ Hospital, James’ Street, Dublin, Ireland; 3 Mater Private Hospital, Eccles Street, Dublin, Ireland

**Keywords:** Lynch syndrome, Colorectal Cancer, DNA mismatch repair (MMR), Database, Microsatellite instability (MSI)

## Abstract

**Background:**

Lynch syndrome (LS) is an autosomal dominant hereditary cancer syndrome responsible for 2–4% of hereditary colorectal cancers (CRC). Mismatch repair protein deficiency (dMMR) is a characteristic feature of LS. It has been associated with a poor response to standard chemotherapy in metastatic colorectal cancer (mCRC). There is currently no LS database to monitor trends of disease in Ireland. We aim to centralise LS data in Ireland to assess the burden of LS in Ireland and guide improvements in prevention and treatment of LS-associated cancer.

**Methods:**

A retrospective review was carried out including all medical records for LS patients from two of the three cancer genetics clinics in Ireland between 2000 and 2018 was carried out. Clinicopathological data of probands (*n* = 57) and affected family members including demographics, mutation status, cancer diagnosis and outcome was recorded. Statistical analysis was carried out using SPSS software.

**Results:**

Fifty-seven families including three-hundred and forty-five individuals affected by cancer were identified. The most common cancers recorded were colorectal (53%), breast (12%) and endometrial (10%). One-hundred and thirty-eight confirmed carriers were identified: 65 *path_MLH1* (47%), 43 *path_MSH2* (31%), 11 *path_MSH6* (8%), 17 *path_PMS2* (12%) and two *path_EPCAM* (1%). Cancer type varied significantly by gene. Median age of first diagnosis was 44.5 years (range 23–81). Half of all deceased patients (*n* = 11) in this group died within 2.5 years of first diagnosis. These deaths were directly related to cancer in 59% of cases.

**Conclusions:**

Under diagnosis of LS misses a powerful preventive and therapeutic opportunity. LS causes early onset dMMR cancer diagnoses with substantial societal impact. Implementation of ICBs into treatment policy for this small cohort of dMMR mCRC is an achievable therapeutic goal that may significantly improve survival. A prospective database for LS in Ireland is necessary to maximise prevention in this population.

## Introduction

Lynch syndrome (LS) is an autosomal dominant hereditary cancer syndrome responsible for 2–4% of hereditary colorectal cancers (CRC) [1, 2]. CRC is the most commonly diagnosed LS-associated cancer in Ireland responsible for 2, 775 new cases diagnosed each year [3]. There is currently no database for LS in Ireland to identify trends in disease. Lack of centralised data for LS in Ireland is a significant barrier to the development of specific guidelines for novel preventative and therapeutic opportunities. Lifetime risk of developing CRC in LS has been reported to be as high as 50–80% depending on gene mutation [1, 4, 5]. Other associated malignancies include endometrial, gastric, ovarian, small bowel, central nervous system and urological cancers [1]. LS-associated cancers develop as a result of pathogenic mutations in one of five genes necessary for mismatch repair (MMR): *MLH1, MSH2, MSH6, PMS2* and *EPCAM* [6, 7]. Impaired ability to correct erroneous strands of DNA results in microsatellite instability (MSI), a phenotypic hallmark of LS-associated CRC [6]. In Ireland, patients with a new diagnosis of LS-associated cancer are assessed for LS risk using the revised Amsterdam and Bethesda criteria. Patients that meet these criteria then have immunohistochemistry (IHC) testing or MSI testing [8]. MSI testing is not part of the universal colorectal tumour testing in Ireland. Following diagnosis of lynch syndrome, surveillance measures are completed in line with NCCN guidelines. If a proband with a pathogenic MMR mutation is identified, they are given an information letter to disseminate to family members. Family members can then avail of predictive testing upon referral by their GP. Identification of MMR deficiency (dMMR) and MSI has additional preventative and therapeutic implications for affected individuals. dMMR cancers have historically displayed a poor response to chemotherapy in the metastatic setting [9–11]. Immune checkpoint blockers (ICB) offer a promising therapeutic alternative for patients with dMMR mCRC. ICBs are currently unavailable for mCRC in Ireland. The aim of this study was to construct and analyse a database of Irish MMR mutation carriers to assess the LS-associated cancer burden in Ireland and identify potential preventative and therapeutic opportunities.

## Methods

### Study design

This study was a retrospective analysis of all LS patients who attended the Mater Misericordiae University Hospital (MMUH)/ Mater Private Hospital (MPH) and St. James’s Hospital (SJH) cancer genetic services in Ireland between 2000 and 2018. Hospital records including patient charts, genetic reports, histopathology reports and family pedigrees were included. A database of all confirmed MMR mutation carriers (affected and unaffected), obligate carriers and affected relatives was collated.

### Participants

During the time period of this study genetic testing services in Ireland were based in Our Lady’s Children’s Hospital Crumlin, SJH, MMUH and MPH. Clinicopathological data including dates of birth, date of death if applicable, cancer diagnosis, age at diagnosis and outcome were recorded from 57 LS pedigrees. Non-specific information recorded from probands regarding possible cancer in family members was omitted if confirmatory documentation could not be identified. Initiall, all affected patients in LS families were recorded. Obligate carriers were identified as patients who must carry the gene mutation based on the autosomal dominant mode of inheritance of LS and the affected members in their family. Affected relatives at 50% risk of carrying the familial MMR mutation were not included in gene-specific analysis as their carrier status remained undetermined. The population was refined to include only those with confirmed LS based on MMR mutation for age, cancer type and outcome analysis.

### Data collection

All cancer diagnoses were first identified according to information recorded on the family pedigree. These were then confirmed by histopathology reports and information from patient records. Gene type, coding change, location and protein were recorded from 99 mutation carriers. Twenty-one cases did not have specific mutation information recorded (12 MLH1, eight MSH2 and one MSH6 mutation). Deaths and cause of death were confirmed using death certificates obtained from the national registry in Ireland. Cancer stage was recorded using information from the histopathology reports and staging scans. When stage was not recorded on histopathology reports, information was gathered from The National Cancer Registry of Ireland (NCRI). This information was only available for deceased patients diagnosed with cancer after 1996.

### Data analysis

Statistical analysis was carried out using SPSS software. Significance was determined by a *P*-value of less than 0.05. An ANOVA (Analysis of variance) test was carried out to evaluate significance of gene-specific variation in age of first diagnosis. For patients with multiple cancers, only the age at first diagnosis was included. Variants of Unknown Significance (VUS) were not included in gene-specific analysis. One-hundred and forty mutation-associated cancers (five unconfirmed) were grouped by genetic mutation: *path_MLH1*, *path_MSH2*, *path_MSH6*, *path_PMS2.* The gene-specific median age of diagnosis of any cancer was then displayed on a box plot using excel.

## Results

### Patient identification

Three-hundred and forty-five affected individuals (300 and 82 cancers in total) affected with cancer were recorded from the family history review of 57 pedigrees. One-hundred and thirty-eight (78 males, 60 females) confirmed MMR mutation carriers were identified, including 97 affected carriers and 41 currently unaffected carriers. The two hundred and seven individuals that were affected by LS-associated cancers within a known LS family had not been tested for LS. Obligate carriers (*n* = 7) were also included in gene-specific analysis. Nine family members of probands tested negative on predictive testing, all of whom were unaffected.

### Patient genotype

Among the 100 and 38 mutation carriers, 65 *path_MLH1* (47%), 44 *path_MSH2* (31%), 12 *path_MSH6* (8%), 17 *path_PMS2* (12%) and two EPCAM (1%) mutations were identified. The number of female and male mutation carriers was 60 (43.8%) and 77 (56.2%) respectively. In this study, we identified three families with the same *path_MLH1* mutation (*path_MLH1* mutation [c.350C > T (p. Thr117Met)]) in exon 4 and three other families with an identical *path_PMS2* mutation [c.137G > T p.(Ser46Ile)]. Three VUS were identified. The MLH1 c.589-9_589-6delGTTT mutation was reviewed by an expert panel in 2013 and found to be of uncertain significance based on research by the International Society for Gastrointestinal Hereditary Tumours (InSiGHT) [12]. VUS in *path_MSH2* were identified in two patients and two cases of constitutional permutation were also recorded in *path_MLH1* genes. (See Table 1) The MSH2 c.913G > A (p.Ala305Thr) mutation was found to be of uncertain significance in 2018 by clinical testing based on Mendelics Assertion Criteria [13]. The MSH2 c.2131_2133delCGA (p.Arg71del) mutation was recorded on IHC reporting as VUS but could not be identified on international databases for MSH2 mutation variants in LS.

**Table 1:** List of Irish MMR mutations recorded in carriers

Gene	MMR mutation	Clinical significance	Location	No. of families with mutation	Total no. of confirmed carriers
MLH1	c.1731 + 1G > A	Pathogenic	NR	1	5
MLH1	c.1928_1931dupTTGA (p.Asn645)	Pathogenic	NR	1	5
MLH1	c.544A > G (p.Arg182Gly)	Pathogenic	Exon 6	1	11
MLH1	c.350C > T (pThr117Met)	Pathogenic	Exon 4	2	6
MLH1	c.1664 T > C (p.Leu555Pro)	Pathogenic	Exon 14	1	1
MLH1	c.589-9_589-6delGTTT	Variant of unknown significance (2013 InSiGHT)	Intron 7	1	1
MLH1	c.735delC (p.Tyr245)	Pathogenic	Exon 9	1	1
MLH1	c.94delA	Pathogenic	Exon 1	1	2
MLH1	C.84_86dupAGC p. (Ala29dup)	Pathogenic	Exon 1	1	1
MLH1	c.84_86dupAGC (p.Ala29dup)	Pathogenic	Exon 1	1	1
MLH1	IVS15 + 1G > A	Pathogenic	NR	1	3
MLH1	deletion	Pathogenic	exons 3–6	1	1
MLH1	c.1937A > G (p.Tyr646Cys)	Pathogenic	exon 17	1	1
MLH1	deletion	Pathogenic	exon 6–8	1	1
MLH1	c.1990-1G > C	Pathogenic	exon 18	2	3
MLH1	IVS17 + 5 > A	Pathogenic	NR	1	1
MLH1	c.1943C > T (p.Pro648Leu)	Pathogenic	exon 17	1	2
MLH1	366-69delAAAG	Pathogenic	exon 4	1	5
MLH1	Hypermethylation	Pathogenic	MLH1 promoter region	1	1
MLH1	Constitutional epimutation	Pathogenic	MLH1 promoter region	1	1
MSH2	c.1684G > T (p.Glu562X)	Pathogenic	NR	1	4
MSH2	c.1786_1788delAA (p.Asn596del)	Pathogenic	exon 12	1	1
MSH2	IVS5 + 3A > T deletion	Pathogenic	NR	2	2
MSH2	c.2251G > A (p.Gly751Arg)	Pathogenic	exon 14	2	4
MSH2	p.A305T:GCA > ACA	Variant of uncertain significance (InSiGHT 2018)	NR	1	1
MSH2	c.2131_2133delCGA (p.Arg71del)	NR	NR	1	1
MSH2	CAT>TAT codon 639 His>Tyr	Pathogenic	NR	1	3
MSH2	2370insT	Pathogenic	exon 14	1	4
MSH2	L277X deletion	Pathogenic	NR	1	1
MSH2	c.3261delC (p. Phe1088Serfs”2)	Pathogenic	NR	1	1
MSH2	c.1277-1G > C	Pathogenic	exon 8	1	1
MSH2	c.1738_1741delGAAA (p.Glu580Leufs*9)	Pathogenic	exon 11	1	1
MSH2	intron 12 rearrangement	Pathogenic	intron 12	1	1
MSH2	c.212-1G > A	Pathogenic	NR	1	1
MSH2	heterozygous deletion	Pathogenic	exon 1–8	2	3
MSH2	c.2050delG (p.Val684)	Pathogenic	NR	1	1
MSH2	c.942 + 3A > T	Pathogenic	intron 5	1	1
MSH2	deletion S743X	Pathogenic	NR	1	1
MSH2	c.342 + 3A > T	Pathogenic	exon 5	1	4
PMS2	c.137G > T (p.Ser46Ile)	Pathogenic	exon 2	4	14
PMS2	deletion	Pathogenic	exon 9–15	1	1
PMS2	deletion	Pathogenic	exons 6–8	1	1
PMS2	deletion	Pathogenic	exons 1–15	1	1
MSH6	deletion	Pathogenic	exons 1–6	1	3
MSH6	c.770dupA(p.Asp257GlufsX6)	Pathogenic	exon 4	1	1
MSH6	3702-3702insAGAA	Pathogenic	NR	1	1
MSH6	c.334C > T (p.Gln132X)	Pathogenic	exon 2	1	1
MSH6	c.2061 T > A (p.Cys687X)	Pathogenic	exon 4	1	2
MSH6	c.2974G > T (p.(GLU992”))	Pathogenic	NR	1	2
MSH6	c.341dupG p. (Lys114GInfs”240)	Pathogenic	NR	1	1
EPCAM	EX6_3’UTRdel	Pathogenic	NR	1	2

*NR* not recorded

### Age characteristics

**Fig. 1:**
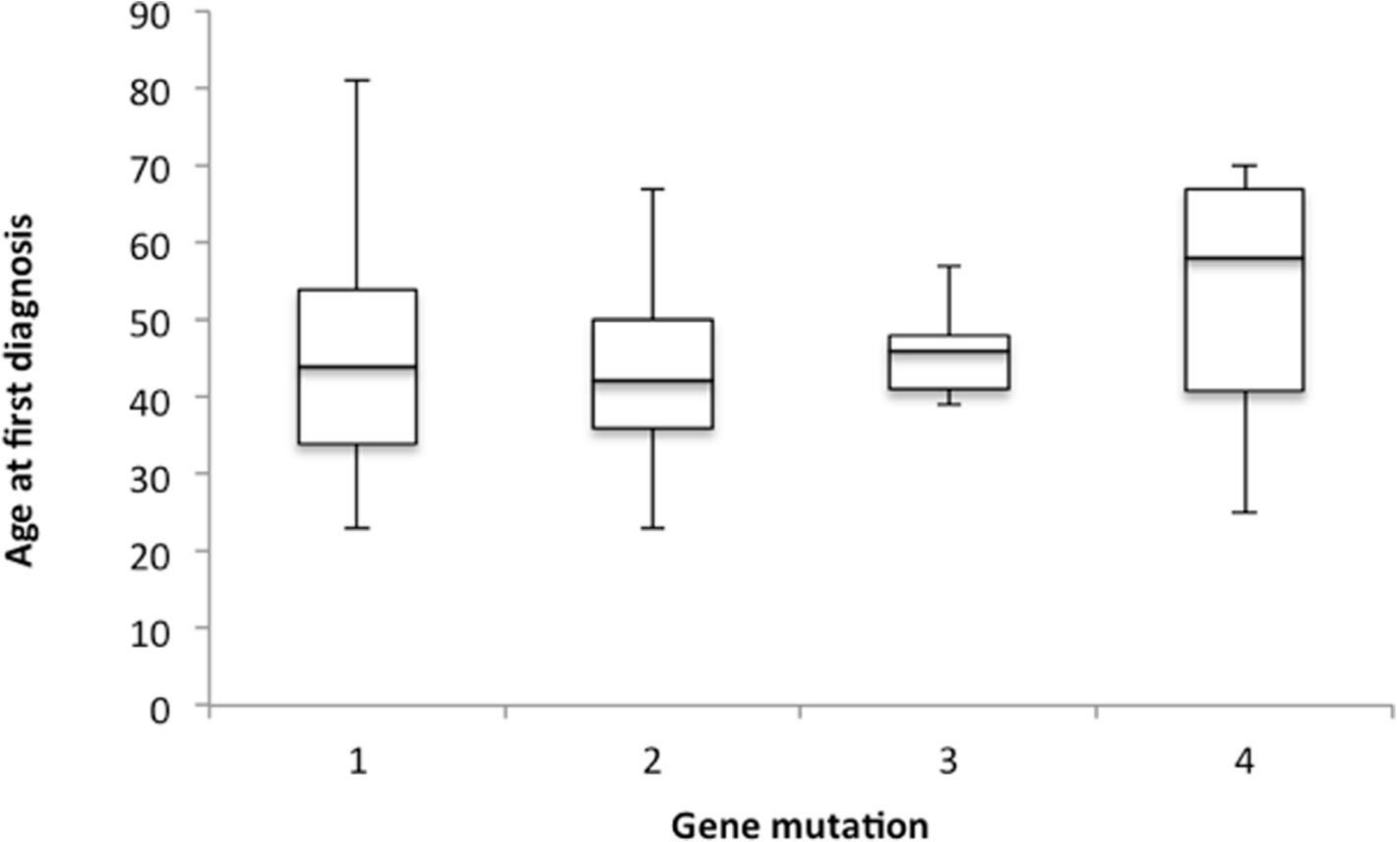
Gene specific age at diagnosis of any LS-associated cancer

Median age of first diagnosis of any LS-associated cancer was 44.5 years (range 23–81) compared to that of Ireland’s general population (65–69 years) [3]. Gene-specific median age at diagnosis of any primary cancer was 44, 42, 46 and 58 years for *path_MLH1*, *path_MSH2*, *path_MSH6* and *path_PMS2* respectively. (see Fig. 1) Although there was a later median age of onset found in *path_PMS2* mutations compared to *path_MLH1*, *path_MSH2* and *path_MSH6*, these results were not found to be statistically significant. (*p* = 0.385).

### Cancer type

**Fig. 2:**
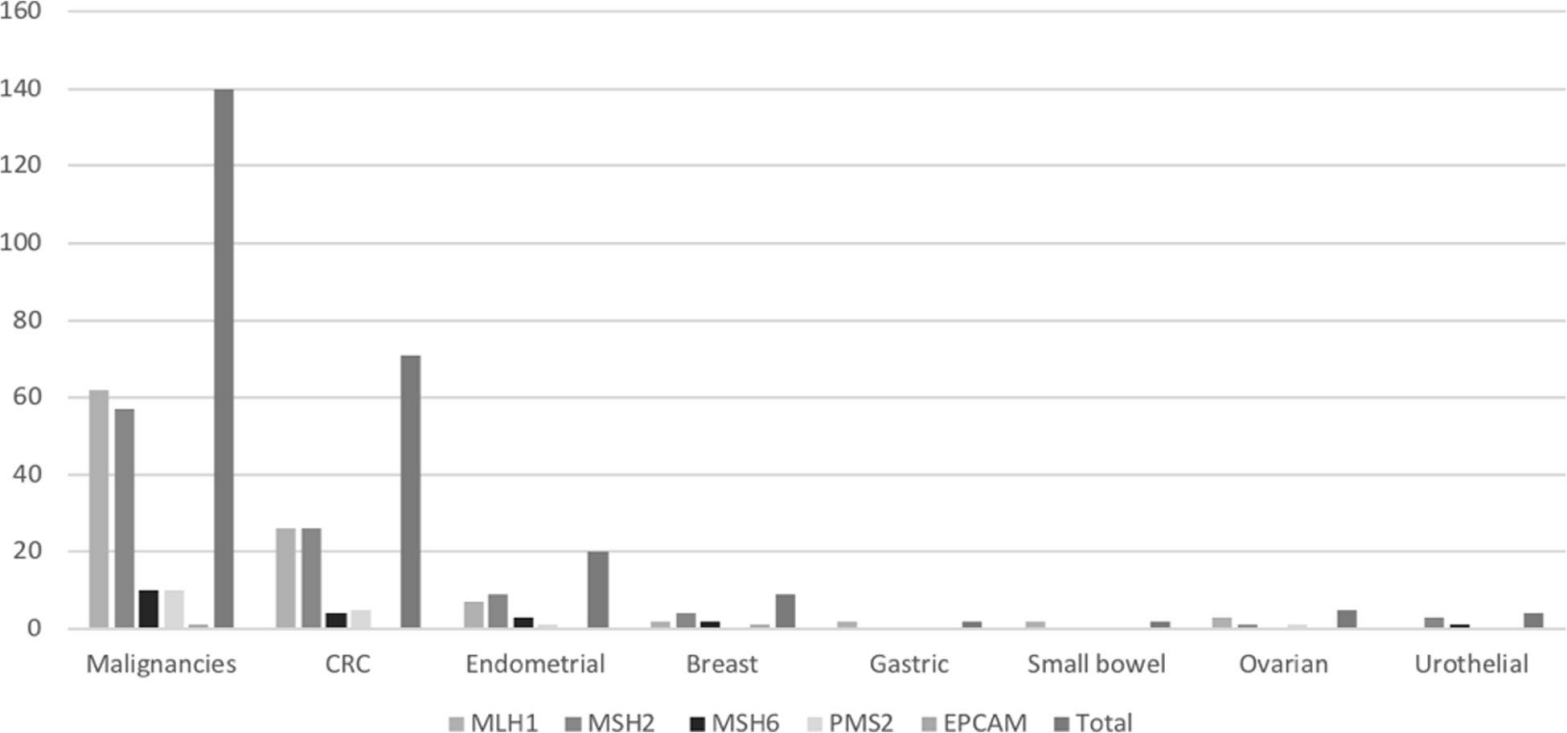
Cancer frequency based on genotype

The three most common cancers identified in the database including all affected family members were colorectal (*n* = 184), breast (*n* = 41) and endometrial (*n* = 34) cancer. (see Fig. 2) Fifty-eight affected individuals had multiple cancer diagnoses. Among mutation carriers, 100 and 40 tumours were recorded in the 97 affected patients. The total number of CRC identified was 71 (50% *path_MLH1*, 36% *path_MSH2*, 6% *path_MSH6*, 8% *path_PMS2*) as well as 20 endometrial, nine breast, five ovarian, four urothelial (75% *path_MSH2*) and two small bowel cancers. (see Table 2) Other single cases included cancers of the stomach, thymus gland, pancreatic, bile duct and a sebaceous neoplasm. CRC was the most common diagnosis across all mutation groups. CRC accounted for 58% of *path_MLH1* associated cancers (*n* = 36), 46% of *path_MSH2* (*n* = 26), 40% *path_MSH6* (*n* = 4) and 50% of *path_PMS2* mutation associated cancers (*n* = 5). Endometrial, breast and urothelial cancers were most commonly identified among *path_MSH2* mutation carriers.

**Table 2:** Cancer frequencies by genotype

Mutation	Affected	Malignancies	CRC	Endometrial	Breast	Small bowel	Ovarian	Urothelial	Other
***path_MLH1***	48	62	36	7	2	2	3	0	12
***path_MSH2***	32	57	26	9	4	0	1	3	14
***path_MSH6***	8	10	4	3	2	0	0	1	0
***path_PMS2***	8	10	5	1	0	0	1	0	3
***path_EPCAM***	1	1	0	0	1	0	0	0	0
**Total**	97	140	71	20	9	2	5	4	29

### LS survival outcomes

Twenty-two (16%) of the mutation carriers were deceased at the time of study commencement, including three probands and seven obligate carriers. Follow up of these patients was up to 15 years depending on age at diagnosis and time from diagnosis death. Cause of death was confirmed by death certificate in 19 of 22 patients. Although a relatively small number of mutation carriers have died, 59% of these deaths were directly related to cancer (*n* = 14). Other causes of death included three cases of pneumonia, one haemorrhagic stroke, and one case of end stage dementia. In three cases the cause of death was unconfirmed. Death occurred within 2.5 years of first diagnosis in 50% of cases. Of all confirmed carriers, 9% (*n* = 13) died within 5 years of diagnosis. Many of these patients had CRC as their primary diagnosis (68%, *n* = 15). All deceased carriers had at least one previous cancer diagnosis and 50% had multiple cancers. In total, 41 tumours were identified in this subgroup. This included colorectal (*n* = 15), endometrial (*n* = 6), breast (*n* = 4), pancreatic (*n* = 2), oesophagus (*n* = 2), small bowel (*n* = 2), splenic (*n* = 2), skin (*n* = 2) and single cases of lymphoma, prostate, ovarian, urothelial, renal and gastric tumours. Overall five-year mortality rate of MMR deficient CRC patients (*n* = 71) was 10% (*n* = 7). Survival time varied significantly by stage. (*p* = 0.048). Stage IV cancer at diagnosis was recorded in six of the deceased patients (*n* = 22).

## Discussion

A notable number of individuals affected by LS in Ireland die from the associated malignancies. A major outcome of this study was recognition of the potential mortality risk in LS. LS is commonly associated with early stage cancer diagnosis and good prognosis leading to the presumption that it does not often lead to cancer-related death [1, 2]. Although most of the patients from this cohort are still alive, 59% of deaths in this population were attributed to cancer. Of this group CRC was unsurprisingly the most common cancer given the large proportion of CRC in LS-associated cancer. All of these patients had dMMR metastatic cancer at time of death. Implementation of ICBs into treatment policy in this cohort is an achievable therapeutic goal that may significantly improve survival. No patients in the group were treated with immunotherapy. In patients with dMMR metastatic colorectal (mCRC) immunotherapy has shown significant improvements in survival compared to standard chemotherapy [14, 15]. PD-1 inhibition offers a promising therapeutic alternative for LS patients with MSI-H tumours [16]. The Keynote 0177 trial recently demonstrated a favourable progression free survival (16.5 vs. 8.2 months) and overall survival (13.7 vs 10.8 months) with use of Pembrolizumab in MSI-H mCRC compared with standard chemotherapy [17]. As part of the ongoing CheckMate142 trial, the combination of CTLA-4 and PD-1 inhibitors in previously treated dMMR MSI-H CRC demonstrated a favourable progression free and overall survival rate at 12 months follow up (71, 85% respectively) [18]. More recently nivolumab and ipilimumab trialed as first line treatment of dMMR MSI-H CRC were found to be well tolerated and demonstrated a 60% overall response rate A major factor in the introduction of these therapies is the relative expense compared to standard treatments. Despite the initial expense, the long-term reduction in morbidity associated with reduction in off-target toxicities may result in an overall favourable cost-benefit ratio. Previous studies have demonstrated cost effectiveness for PD-1 inhibitors used in other cancer types [19]. Meticulous patient selection has been suggested as the most important consideration in ensuring cost-effectiveness [19]. A national effort must be made to consider the value of these molecular therapies in LS patients with metastases in the future.

A dedicated infrastructure is required to maximise the potential of prevention in this cohort of individuals. The demand on screening services in Ireland has increased with the rise in incidence and awareness of CRC. A dedicated LS screening service could be established by adaptation of current screening protocols for CRC [8]. Due to the early age of onset in most LS-associated cancer, patients often develop cancers before the age of qualification for national screening programmes [1]. In this study, 41 mutation carriers were unaffected by cancer at the time of the study. NCCN guidelines are currently used to guide Irish LS surveillance regimens. Surveillance by colonoscopy at 20 to 25 years or 2–5 years before onset of the earliest cancer is the current recommendation [8, 20]. Three-yearly colonoscopy screening had previously been shown to reduce mortality from LS-associated CRC by up to 65% [21]. British guidelines in now recommend 2-yearly colonoscopy with age for initial screening varying by gene [22]. Recent reviews by the Prospective Lynch Syndrome database (PLSD) found that further reducing the interval between colonoscopies did not reduce the incidence of CRC nor the stage at diagnosis [23]. Colonoscopic screening at appropriate intervals is still a vital measure alongside clinical surveillance to prevent potentially fatal malignancy [24]. The importance of early recognition must also be considered for extra-colonic cancer in LS. Endometrial cancer is the second most common LS-associated cancer. Screening has not demonstrated survival benefit for women with LS to date [20]. Screening for endometrial cancer is only recommended after the age of 35-40 years in MLH 1 MSH2 MSH6 cancer according to a recent study by the Manchester consensus [25]. There is also no screening protocol for breast cancer in LS as it has not been widely recognised as a LS-associated cancer. This study has shown that breast cancer is the third most common to LS associated cancer in Ireland. Some studies have previously highlighted the link between MMR gene mutations and breast cancer [26]. *path_MLH1* has been suggested as the highest risk gene for breast cancer in LS with a cumulative risk of developing breast cancer by 70 years reported as high as 18.6% [27]. In our study *path_MSH2* carried the largest proportion of breast cancers. The lower median age of diagnosis (51 years) compared to 60–64 years in the general population supports the possibility of a genetic component. Breast cancer is the most commonly diagnosed cancer in females in Ireland (incidence rate = 121.6) [3]. The large proportion of breast cancer in this group may therefore be incidental. It is important to consider breast cancer as a possible LS-associated cancer with thorough clinical review rather than implementation of an earlier screening programme in this group. It is currently recommended that for LS patients, screening decisions be individualised by family history with usual screening commencing 5–10 years before the youngest breast cancer diagnosis in the family [28].

This database highlights the potential under diagnosis of LS and a missed opportunity for preventable cancer in Ireland. Databases have been used internationally since the 1980s in countries such as Finland, Denmark and Sweden. Data has been used to highlight disease trends and identify the distribution of cancer type, cumulative risk of cancer and outcome in LS [29–31]. The InSiGHT database was the first database for identification of pathogenic variants contributing to LS. In 2012 the PLSD was founded by members of InSiGHT previously known as the Mallorca group, now the European hereditary tumour group. This group includes data from nine different countries including Finland, Netherlands, Spain, Germany, Britain, Israel and Australia. PLSD has open access to a website which allows individuals to determine their cumulative risk of cancer based on specific genetic variants in each organ depending on age and gender. From this cohort, nine cases were confirmed in 2016, three in 2015 and three in 2014. Given the estimated contribution of LS to hereditary CRC with reported prevalence of LS as high as 1:100–1:180, the number of confirmed carriers in this database may be an underrepresentation of the total LS population in Ireland [32, 33]. Under-diagnosis of LS misses a powerful preventative and therapeutic opportunity. Many countries have developed prospective databases for LS in their country to prevent this [32]. There is currently no database for LS in Ireland. A national database would facilitate appropriate resource allocation for a high-risk screening service and enable prospective follow-up and monitoring of service utilisation. Each newly confirmed patient in Ireland with MMR mutation either affected or unaffected could be prospectively added to a single national database based in one of three genetic centres in Ireland. Based on PLSD relevant factors to be recorded for each patient should include age, gender, age at diagnosis, age at death and the path_MMR mutation identified [34]. Identification of specific gene variants for analysis of mutation pathogenicity can also be achieved through database analysis [35]. Patient education in LS facilitates a collaborative approach in future management decisions. Although formation of a LS database in itself is not a novel idea, the data collected retrospectively from this database allows for identification of the frequency of pathogenic variants in this country and subsequent comparison with other international databases. Identification of trends in the natural history of Irish LS cases in comparison with other countries will allow for further collaboration in the worldwide LS community. Due to the relatively small population in Ireland, identification of all patients with LS and subsequent addition to the database is an achievable opportunity for optimum surveillance.

This study has demonstrated the burden of LS in Ireland. Data collection was limited by the inclusion of LS patients from only two of three genetic clinics in Ireland. The under-diagnosis of LS may have also contributed to the low number of LS patients identified in the study. A more comprehensive review of Irish LS patients with a larger sample population would improve the external validity of these results. Prognostic indicators such as co-morbidities and treatment variation between groups were not taken into consideration in these patients. These factors must be addressed to identify the true risk of mortality with LS-associated cancers.

In conclusion, LS is an under recognised cause of CRC in Ireland and carries a significant burden of disease for many Irish LS kindreds. This study documented trends in age, genotype, cancer type and overall outcome in the Irish LS population. We recommend implementation of immunotherapy as a treatment option for LS patients in Ireland with mCRC. Early diagnosis and colonoscopic surveillance commencing between 20 and 25 at 3 yearly intervals is recommended in accordance with NCCN guidelines. Surveillance for endometrial and breast cancer should be considered on a case by case basis following thorough clinical review. Following this study, we aim to implement a prospective LS database at local and national level to monitor trends in our LS population. A national LS database and universal diagnostic, screening and therapeutic approach would maximise the potential of prevention in this population.

## Data Availability

All data and materials including software application used complied with field standards. Data may be requested from Alice Talbot if required.
